# Impact of intensive care unit admission on the resumption of treatment in patients with cancer: a systematic review and meta-analysis

**DOI:** 10.1007/s00520-026-11135-6

**Published:** 2026-08-31

**Authors:** Raisa do Val Roso, Renato Daltro-Oliveira, Laveena Munshi, Antonio Paulo Nassar Junior

**Affiliations:** 1 Intensive Care Unit, AC Camargo Cancer Center, São Paulo, Brazil; 2 Department of Medicine, Interdepartmental Division of Critical Care Medicine, Sinai Health System, University of Toronto, Toronto, Canada

**Keywords:** Anticancer agents, Cancer, Cancer treatment protocol, Critical care, Disease progression

## Abstract

**Purpose:**

Patients with cancer represent a substantial and growing proportion of the ICU population. Although short-term survival has improved, it remains unclear how often anticancer treatment is resumed after critical illness. We aimed to synthesize the available evidence on treatment resumption following ICU discharge in adult patients with cancer.

**Methods:**

We searched PubMed, Embase, Scopus, and Web of Science from inception to February 24, 2025 (PROSPERO CRD420251080476). We included studies reporting resumption of systemic anticancer treatment after unplanned ICU admission in adults with solid or hematological malignancies. The primary outcome was the proportion of patients resuming any anticancer treatment after discharge. The secondary outcome was resumption of the originally planned treatment.

**Results:**

Twenty studies encompassing 3,551 patients were included. Fifteen studies encompassed patients with solid cancer (*N* = 1800), three patients with hematological cancer (*N* = 261), and two included both solid and hematological malignancies (*N* = 1490). Two studies were multicenter. The pooled rate of resuming any anticancer treatment after discharge was 61% (95% CI, 51–70%), with substantial heterogeneity (I^2^ = 95.6%). Resumption rates were 60% (95% CI, 48–71%) in patients with solid tumors and 77% (95% CI, 56–89%) in patients with hematological malignancies. Eight studies (1,736 patients) assessed resumption of the original planned regimen, with a pooled rate of 48% (95% CI, 36–60%) at 6 months post-discharge.

**Conclusion:**

Most patients surviving ICU admission resume anticancer therapy, but fewer than half continue their originally planned treatment. ICU admission may represent a turning point in the oncologic trajectory, highlighting the need for early post-ICU reassessment of disease status, functional capacity, and patient preferences. However, there is a lack of prospective multicenter studies with standardized definitions of treatment resumption and predefined assessment time points.

**Supplementary Information:**

The online version contains supplementary material available at https://doi.org/10.1007/s00520-026-11135-6.

## Introduction

Over the past decades, advances in cancer diagnosis and treatment have substantially reduced cancer-related mortality and prolonged survival among patients with malignancies [1]. As a result, a growing number of patients with cancer experience acute complications that may be disease- or treatment-induced, necessitating admission to the intensive care unit (ICU) [2]. Recent data indicate that up to 5% of patients with solid tumors and more than 20% of those with specific hematologic malignancies are admitted to the ICU within the first year following diagnosis [3–5]. Currently, approximately one quarter of all patients admitted to the ICU have a concurrent diagnosis of cancer [6]. While short-term outcomes have improved, with hospital survival exceeding 70% in many settings [6, 7], we lack data on their longer-term outcomes.

This evolution in cancer critical care survival has changed the focus to events following ICU discharge, including long-term outcomes of patients with cancer after critical illness. Thus, a central question after ICU discharge is whether anticancer treatments can be resumed. Survivors of critical illness frequently experience persistent physical symptoms [8], functional decline, and psychological distress [9], all of which may interfere with the continuation of anticancer therapy and, consequently, cancer control, cure, and prognosis. Despite the clinical impact of this issue, there are few studies specifically aimed at assessing treatment resumption after ICU admission in patients with cancer [10–12].

Thus, a comprehensive synthesis of the available evidence is needed to better understand how often anticancer treatment is resumed after an unplanned ICU admission and to identify knowledge gaps that may inform clinical decision-making and future research. Therefore, our primary objective was to identify and synthesize the available evidence regarding the resumption of any anticancer treatment among adult patients with cancer who survive an unplanned ICU admission. As a secondary objective, we sought to estimate the pooled prevalence of patients resuming their planned anticancer treatment after ICU discharge.

## Methods

### Study design and reporting

This systematic review and meta-analysis was conducted in accordance with the Preferred Reporting Items for Systematic Reviews and Meta-Analyses (PRISMA) guidelines [13]. The review protocol was prospectively registered in the International Prospective Register of Systematic Reviews (PROSPERO registration number CRD420251080476).

### Eligibility criteria

We included observational studies (prospective or retrospective cohort studies and case–control studies) and clinical trials involving adult patients with solid tumors or hematologic malignancies who were admitted to the ICU on an unplanned basis. Eligible studies were required to report outcomes related to the continuity of anticancer treatment after ICU admission, including any resumption, continuation of the previously planned regimen, modification, or interruption in systemic treatment. Anticancer systemic treatment could include surgery, chemotherapy, radiotherapy, immunotherapy, targeted therapy, hormone therapy, or hematopoietic stem cell transplantation.

We excluded studies conducted exclusively in pediatric populations, studies not involving ICU admission, studies that did not report outcomes related to resuming cancer treatment after ICU discharge, and studies that included only patients with decisions to forgo life-sustaining therapies.

### Search strategy

We conducted a systematic literature search using the following electronic databases: PubMed, Embase, Scopus, and Web of Science from inception to February 24, 2025. The search strategy combined controlled vocabulary and free-text terms related to cancer, intensive care, and continuity of anticancer treatment, with no language, country, or publication date restrictions. The full search strategies for all databases are provided in Table S1.

### Study selection

One reviewer (APNJ) imported all records identified by the search into the Rayyan platform [14] and removed duplicates. Two reviewers (RVS and RDO) independently selected studies, screened titles and abstracts for eligibility, and conducted full-text reviews of potentially relevant articles. Disagreements were resolved through discussion and consensus.

### Data collection

Two reviewers (RVS and RDO) conducted data extraction using a standardized data collection form developed for this review. When necessary, the review team attempted to clarify unclear data through discussion.

For each included study, we extracted information on study characteristics (year of publication, country, study design, number of centers, study period), type of malignancy (solid or hematological), site of the solid cancer or type of hematological malignancy, number of included patients, pre-ICU performance status, severity at ICU admission, main reasons for ICU admission, use of invasive mechanical ventilation, types of anticancer treatments assessed, number of ICU survivors with the reported outcomes related to anticancer treatment after ICU admission, time of assessment of treatment resumption, and risk factors associated with resumption of anticancer treatment. In studies that included patients with both solid and hematological cancers, we contacted the authors to obtain separate data for each cancer type. We defined the resumption of treatment as the initiation of any anticancer treatment after ICU or hospital discharge. We defined the resumption of the original treatment as the administration of the same treatment planned prior to ICU admission. The primary outcome was the proportion of patients who resumed any anticancer treatment after ICU or hospital discharge. The secondary outcome was the proportion of patients who continued the exact same planned treatment before ICU admission.

### Risk of bias assessment

Two reviewers (RVS and APNJ) independently assessed the methodological quality and risk of bias of the included studies using the Joanna Briggs Institute (JBI) critical appraisal tools appropriate for prevalence studies [15]. Disagreements were resolved by consensus. For the overall appraisal, we classified the studies according to the number of domains at the risk of bias: (1) Low: Zero, (2) Moderate: 1 or 2 domains, (3) High: 3 or more domains.

### Statistical analysis

For the quantitative synthesis, the primary measure was the proportion of patients who resumed anticancer treatment. The secondary measure was the proportion of patients who continued the planned treatment before ICU admission. Proportions were pooled using a random-effects model to account for anticipated clinical and methodological heterogeneity among studies. Statistical heterogeneity was assessed using the I^2^ statistic. The magnitude of between-study variance was assessed using the τ^2^. We assessed publication bias through visual inspection of funnel plots and using Egger’s linear regression test. We performed a preplanned subgroup analysis by malignancy type within each study (solid, hematological, or both). Finally, we conducted a secondary analysis by extracting individual cohorts from studies that included both cancer types. This allowed us to combine these data with studies focusing exclusively on either solid or hematological malignancies. All analyses were performed using RStudio (Version 2025.09.1 + 401).

## Results

### Study screening and selection

**Fig. 1:**
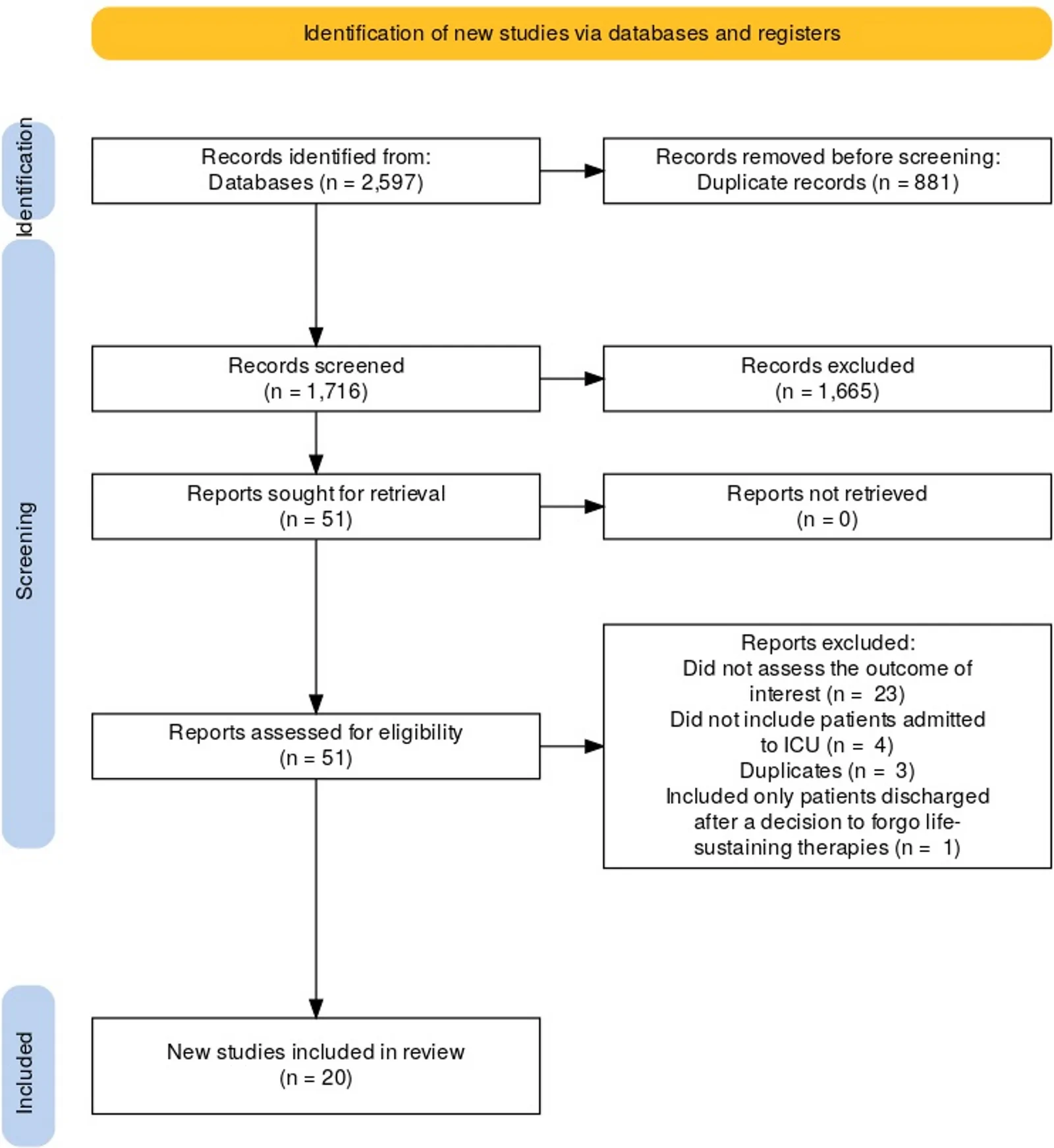
Preferred reporting items for systematic reviews and meta analyses flowchart

Our search retrieved 2597 records, of which 51 underwent full-text review, and 20 were included in this systematic review and meta-analysis [4, 10–12, 16–31]. We excluded one study that included only patients discharged from the ICU after a decision to forgo life-sustaining therapies [32] (Fig. 1).

### Characteristics of included studies

Of the 20 included studies, 12 were conducted in France; two were multicenter, and one was prospective. Fifteen studies included patients with solid tumors, of which 8 included patients with predetermined sites, with lung cancer being the most common (*N* = 4). Three studies included patients with hematological malignancies; one included only patients with acute myeloid leukemia and B-cell non-Hodgkin lymphoma, and the other included only patients with diffuse large B-cell lymphoma. Two studies included both patients with solid and hematological malignancies (Table 1). One study was at low risk of bias; 12 studies were at moderate risk, and 7 were at high risk of bias (Table S2).

**Table 1:** Characteristics of the included studies

Study	Year	Country	Study design	Study period	Type of cancer	Site or type	Number of patients	Time of assessment	Anticancer treatments assessed	Resumption of any anticancer treatment (%)		Resumption of the original anticancer treatment (%)
Roques	2009	France	Single-center retrospective cohort	1997–2006	Solid	Lung	44	After hospital discharge	Chemotherapy, Radiotherapy, surgery	30 (68.2)		NA
Andréjak	2011	France	Single-center retrospective cohort	1996–2006	Solid	Lung	27	After ICU discharge	Chemotherapy, Radiotherapy, Surgery	4 (14.8)		NA
Soares	2014	Argentina, Brazil, Chile, France, United Kingdom, and Uruguay	Multicenter prospective cohort	2011	Solid	Lung	249	At 6 months after discharge	Chemotherapy, Radiotherapy	102 (41.0)		NA
Lee	2015	South Korea	Single-center retrospective cohort	2010–2012	Solid and hematological	Unrestricted	356	After ICU discharge	Chemotherapy, Radiotherapy, Bone marrow transplation	161 (45.2)		NA
Wohlfarth	2015	Austria	Single-center retrospective cohort	2000–2014	Hematological	DLBCL	12	After ICU discharge	Chemotherapy	9 (75.0)		NA
Auclin	2017	France	Single-center retrospective study		Solid (patients aged ≥ 65 years)	Unrestricted	121	After ICU discharge	Chemotherapy, targeted therapy	77 (63.6)		NA
Decavèle	2020	France	Single-center retrospective cohort	2009–2018	Solid	High-grade glioma	78	After ICU discharge	Chemotherapy, Radiotherapy, Corticosteroids	60 (76.9)		45 (57.7)
Epstein	2020	USA	Single-center retrospective cohort	2014	Solid	Gastrointestinal(Metastatic)	15	After hospital discharge	Chemotherapy	6 (40.0)		5 (33.3)
Gheerbrant	2021	France	Single-center retrospective cohort	2005–2013	Solid	Unrestricted	87	At 3 months after discharge	Chemotherapy, Radiotherapy, Surgery, outros	74 (85.1)		NA
Vigneron	2021	France	Single-center retrospective cohort	2007–2018	Solid	Lung	115	After ICU discharge	Chemotherapy, hormone therapy, targeted therapy, surgery	58 (50.4)		NA
Hautecloque-Rayz	2022	France	Single-center retrospective cohort	2014	Solid(Metastatic)	Unrestricted	96	After ICU discharge	Chemotherapy, Surgery	59 (61.5)		NA
Toffart	2023	France	Single-center retrospective cohort	2020–2021	Solid	Lung, colorectal, head and neck	23	At 3 months after discharge	Chemotherapy, immunotherapy, radiotherapy, surgery, targeted therapy	21 (91.3)		16 (69.6)
Vigneron	2023	France	Single-center retrospective cohort	2007–2020	Solid	Breast	76	After hospital discharge	Chemotherapy, immunotherapy, targeted therapy, radiation therapy, surgery	65 (85.5)		NA
Benguerfi	2024	France	Single-center retrospective cohort	2014–2019	Solid	Unrestricted	136	After ICU discharge	Radiotherapy, Chemotherapy, Immunotherapy, Targeted therapy, hormoniotherapy		81 (59.6)	62 (45.6)
de Herreros	2024	Spain	Single-center retrospective cohort	2019	Solid	Unrestricted	64	After hospital discharge	Chemotherapy	25 (39.1)		NA
Lee	2024	South Korea	Single-center retrospective cohort	2016–2022	Solid	Unrestricted	568	After ICU discharge	NA	246 (43.3)		NA
Vizzacchi	2024	Brazil	Single-center retrospective cohort	2015–2018	Solid and hematological	Unrestricted	1134	After hospital discharge	Chemotherapy, Radiotherapy	905 (79.8)		636 (70.3)
Bredin	2024	France	Single-center retrospective cohort	2013–2021	Hematological	AML and B-NHL	170	After ICU discharge	NA	131 (77.1)		98 (57.6)
Munshi	2024	Canada	Multicenter retrospective cohort	2018–2020	Hematological	Unrestricted	79	At 6 months after discharge	Chemotherapy, immunotherapy, targeted therapy, radiation therapy, hormone therapy, surgery, bone marrow transplatation	38 (48.1)		20 (25.3)
Ferrari	2025	France	Single-center retrospective cohort	2005–2018	Solid	Unrestricted	101	After ICU discharge	NA	40 (39.6)		26 (25.7)

AML: Acute Myeloid Leukemia. B-NHL: B-cell Non-Hodgkin Lymphoma. DLBCL: Diffuse Large B-Cell Lymphoma. NA: Not available

The 20 included studies comprised 5948 patients, of whom 3551 were assessed for treatment resumption after discharge. The number of patients assessed for treatment resumption ranged from 12 to 1134 (Table 1). The minority of patients had poor performance status at admission; the main reasons for ICU admission were infection/sepsis and acute respiratory failure; and the proportion of patients requiring mechanical ventilation during ICU admission ranged from 8.8 to 67.7% (Table 2). Definitions of anticancer treatment resumption (Table S3) and the time point of assessment varied across studies (from at ICU discharge to 6 months after discharge) (Table 1). There was no evidence of significant publication bias in the pooled estimates of the resumption of treatment rate (*p* = 0.91) (Figure S1).

**Table 2:** Characteristics of the patients in the included studies

Study	Year	Total number of patients in the ICU cohort		Cohort data extraction		Poor performance status, N (%)		Severity at ICU admission		Main reasons for ICU admission, N (%)			Invasive mechanical ventilation,N (%)
Roques	2009	105		All patients		PS 2–4 = 47 (44.8)		SAPS II, mean (SD) = 40 (21)SOFA, mean (SD) = 4.4 (4.7)		Acute respiratory failure = 62 (59.0), hemoptysis = 47 (44.8), septic shock = 10 (9.5)			43 (41)
Andrejak	2011	76		ICU survivors (*N* = 40)		NA		NA		Noninfectious pulmonary disorders = 18 (45.0), Infection = 15 (37.5)			19 (47.5)
Soares	2014	449		Hospital survivors (*N* = 203)		PS 3–4 = 8 (3.9)		SAPS II, mean (SD) = 36.5 (13.9)SOFA, median (IQR) = 4 (2–6)		Cancer-related complications = 79 (38.9)			79 (38.9) on day 1
Lee	2015	525		All patients		PS > 2 = 25 (4.8)		SAPS 3, median (IQR) = 78 (67–90)SOFA, median (IQR) = 8 (6–11)		Respiratory failure = 219 (41.7), Severe sepsis or septic shock = 213 (40.6)			216 (41.1)
Wohlfarth	2015	37		ICU survivors (*N* = 28)		NA		SAPS II, median(IQR) = 46 (26–102)		Shock = 10 (35.7), Infection = 11 (39.3), acute respiratory failure = 7 (25.0)			18 (64.3)
Auclin	2017	262		ICU survivors (*N* = 174)		PS 3—4 = 18 (10.3)		SAPS II, mean (SD) = 51.4 (13)		Sepsis = 57 (32.8), acute respiratory failure = 47 (27)			54 (31.0)
Decavèle	2020	91		All patients		KPS (treatment resumption vs. no resumption [median (IQR)] = 70 (60–80) vs. 70 (50–80)		SAPS II, median (IQR) = 32 (18–50)SOFA, median (IQR) = 5 (3–7)		Coma with seizures = 43 (47.3), acute respiratory failure = 18 (19.8), Coma without seizures = 15 (16.5)			45 (49.5)
Epstein	2020	28		All patients		NA		Number of patients with MPM II > 80 = 2 (7.1%)		Sepsis = 11(39.3), respiratory failure = 9 (32.1)			16 (57.1)
Gheerbrandt	2021	361		ICU survivors (*N* = 253)		PS 3—4 = 51 (20.2)		SAPS II, median (IQR) = 46 (36–58)		Not related to cancer = 117 (46.2), Complications of anticancer treatment = 61 (24.1), Tumor progression = 60 (23.7)			103 (40.7)
Vigneron	2021	379		All patients		PS 3–4 = 82 (21.6)		Median SOFA = 4–7 during study years		Infection = 113 (29.8), pleural effusion = 19 (5.0)			174 (45.9)
Hautecloque-Rayz	2022	129		All patients		PS 3 = 8 (6.2)		Three or more organ failures, N (%) = 27 (20.9)		Cancer = 39 (30.2), comorbidities = 29 (22.5)			39 (30.2)
Toffart	2023	23		ICU survivors (*N* = 23)		PS 2–3 = 5 (21.7)		SAPS II, median (IQR) = 43 (33–51)		Respiratory = 13 (56.5), Cardiovascular = 9 (39.1), Sepsis = 3 (13.0)			9 (39.1)
Vigneron	2023	177		All patients		PS 3–4 = 33 (18.6)		SOFA, median (IQR) = 5 (4–8)		Infection = 56 (31.6), pleural effusion = 10 (5.6), hypercalcemia = 10 (5.6)			72 (40.7)
Benguerfi	2024	219		Assessment of anticancer treatment resumption (*N* = 130)		PS 3—4 = 13 (10.0)		SAPS II, median (IQR) = 44.0 (32.8–66.3)		Acute respiratory failure = 41 (31.5), septic shock = 19 (14.6)			56 (43.1)
de Herreros	2024	84		All patients		PS 2–4 = 25 (29.8)		SOFA, mean (SD) = 4.7 (2.8)APACHE II, mean (SD) = 14.5 (5.9)		Respiratory failure = 37 (38.1), sepsis/septic shock = 32 (32.9) *			14 (14.4)
Lee	2024	955		All patients		PS 2–4 = 260 (27.2)		NA		Acute respiratory failure = 441 (46.2), Shock = 280 (29.3)			647 (67.7)
Vizzacchi	2024	1134		Hospital survivors (*N* = 1134)		PS 3–4 = 0 (0)		SAPS 3, mean (SD) = 62.1 (12.1)		Sepsis = 364 (32.1), acute respiratory failure = 167 (14.7), cardiovascular diseases = 157 (13.8)			100 (8.8)
Bredin	2024	366		Assessment of anticancer treatment resumption (*N* = 170)		PS 3—4 = 36 (21.2)		SAPS II, median (IQR) = 42 (31–51)SOFA, median (IQR) = 5 (4–8)		Circulatory failure = 49 (28.8), acute respiratory failure = 33 (19.4), metabolic disturbances = 23 (13.5)			30 (17.6)
Munshi	2024	414		ICU survivors (*N* = 203)		Moderate/severe frailty (i.e., CFS = 6–9) = 41 (20.2)		SOFA, median (IQR) = 8 (5–10)		Acute respiratory failure = 83 (40.9), Sepsis = 83 (40.9), Cardiac complication = 31 (15.3)			59 (29.1)
Ferrari	2025	134		All patients		PS > 1 (assessed in 100 patients), N (%) = 75 (75)		SAPS II, mean (SD) = 50.9 (16.9)APACHE II, mean (SD) = 22.2 (7.4)SOFA, mean (SD) = 6.3 (3.8)		Sepsis = 82 (61.2), tumoral complications = 26 (19.4)			72 (53.7)

APACHE: Acute Physiology and Chronic Health Evaluation. CFS: Clinical Frailty Scale. IQR: Interquartile range. KPS: Karnofsky Performance Status. NA: Not available. PS: Performance status. SAPS = Simplified Acute Physiology Score. SD = Standard Deviation. SOFA = Sequential Organ Failure Assessment

^*^In the study by de Herreros et al., the main reasons for ICU admission were presented based on the total number of admissions (*N* = 97) and not the total number of patients (*N* = 84)

### Primary analysis: Resumption of any anticancer treatment after discharge

**Fig. 2:**
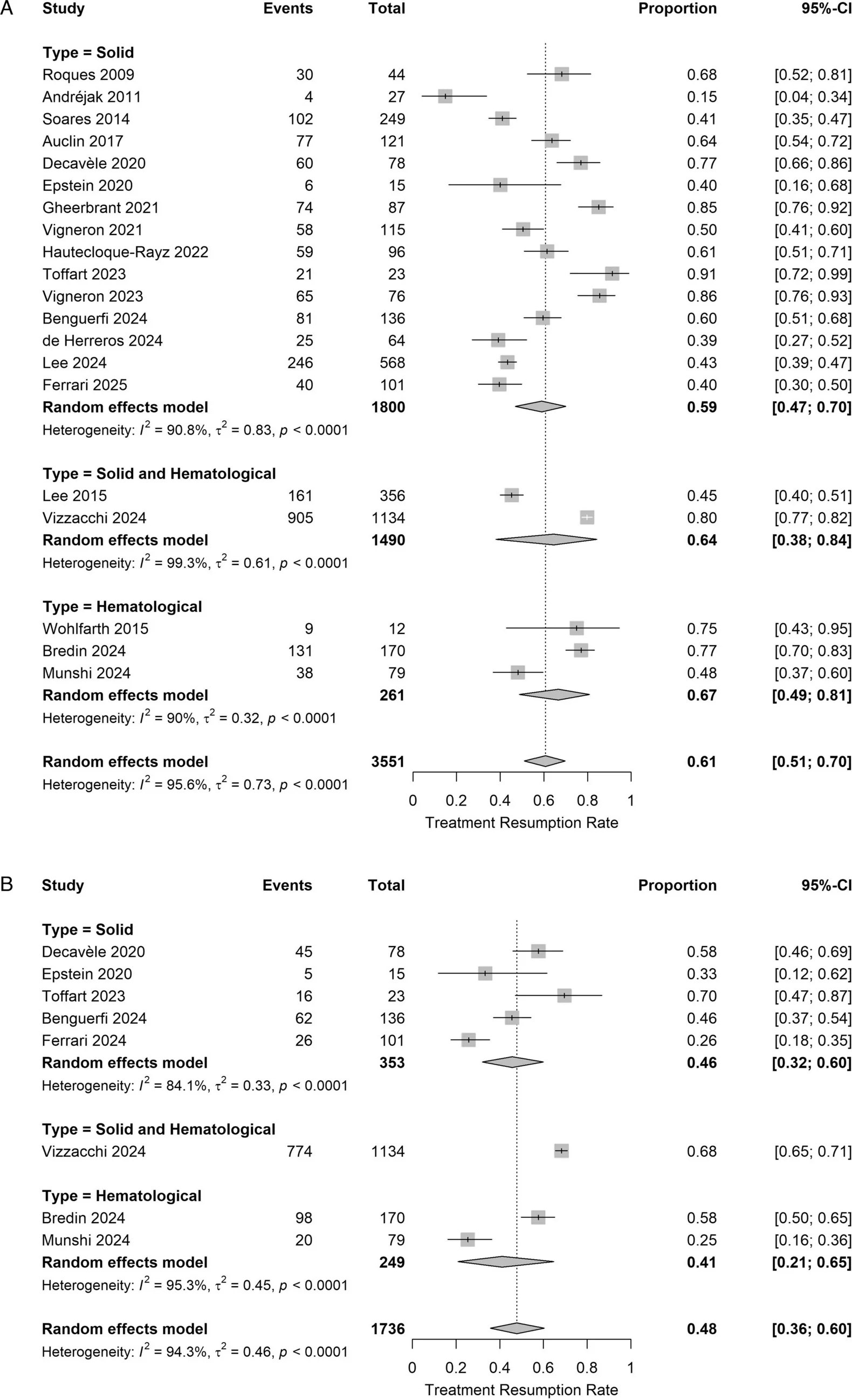
Random-effects forest plot of resumption of any anticancer treatment after ICU admission (**A**) and of resumption of the original anticancer treatment after ICU admission (**B**)

The pooled treatment resumption rate was 61% (95% CI, 51–70%) within 6 months after discharge. Of the 15 studies that included only patients with solid malignancies (*N* = 1800), the treatment resumption rate was 59% (95% CI, 47–70%). The pooled analysis of the three studies, which included only patients with hematological malignancies (*N* = 261), showed a resumption rate of 67% (95% CI, 49–81%). Two studies included patients with both solid and hematological malignancies (*N* = 1490), and their pooled resumption rate was 64% (95% CI, 38–84%). All analyses had substantial heterogeneity (Fig. 2A).

### Secondary analysis: Resumption of the original anticancer treatment after discharge

Eight studies, encompassing 1736 patients, assessed the rate of resumption of the original anticancer treatment. The pooled resumption rate was 48% (95% CI, 36–60%) within 6 months. Of the five studies that included only patients with solid malignancies (*N* = 353), the treatment resumption rate was 46% (95% CI, 32–60%). The pooled analysis of the two studies, which included only patients with hematological malignancies (*N* = 249), yielded a 41% rate (95% CI, 21–65%). The only study that included both solid and hematological malignancies (*N* = 1134) reported a resumption rate of 68% (95% CI, 65–71%) (Fig. 2B).

### Secondary analysis: Resumption of any anticancer treatment according to the type of malignancy

**Fig. 3:**
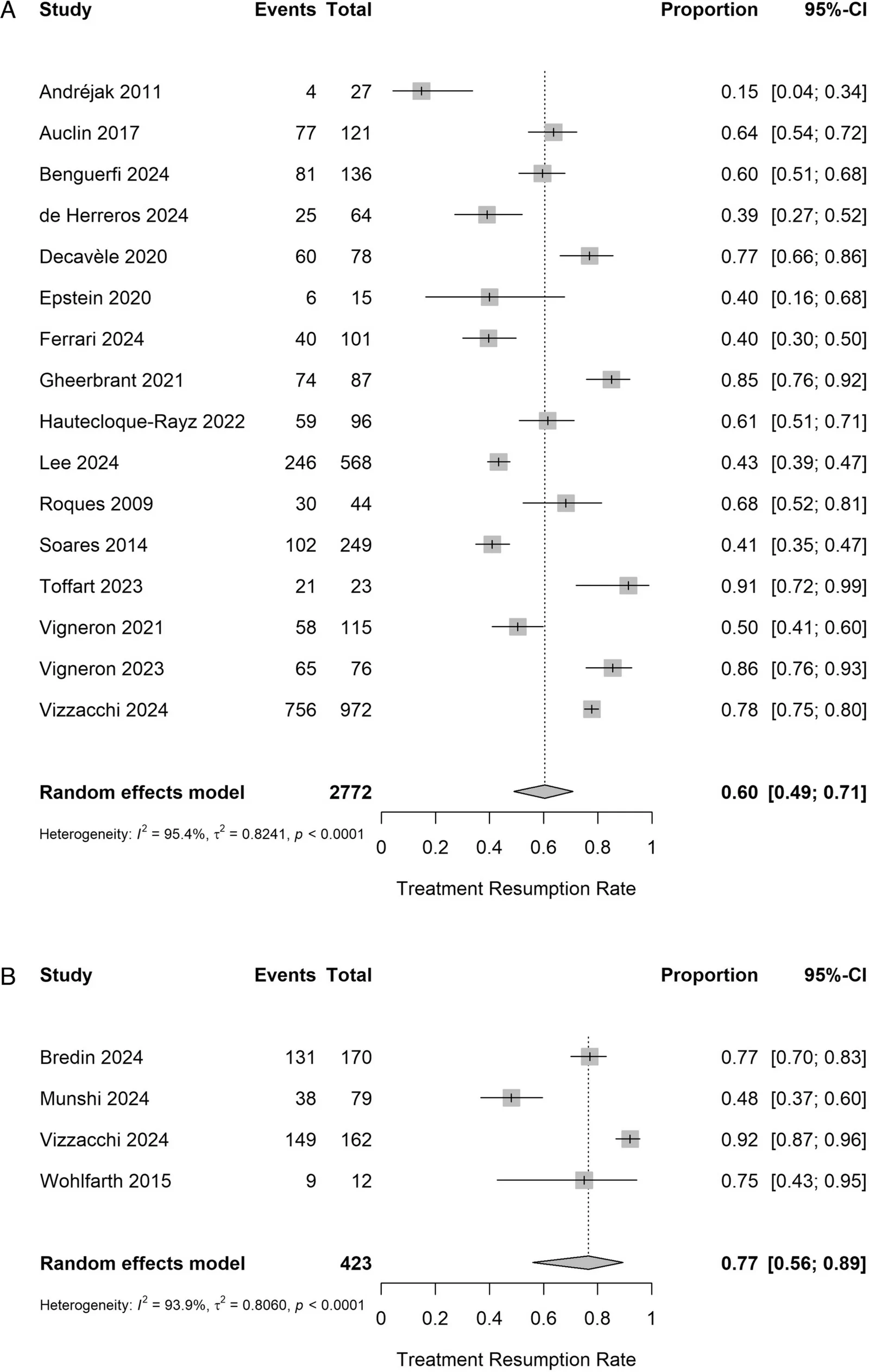
Random-effects forest plot of resumption of any anticancer treatment after ICU admission in patients with solid (**A**) and hematological malignancies (**B**)

**Fig. 4:**
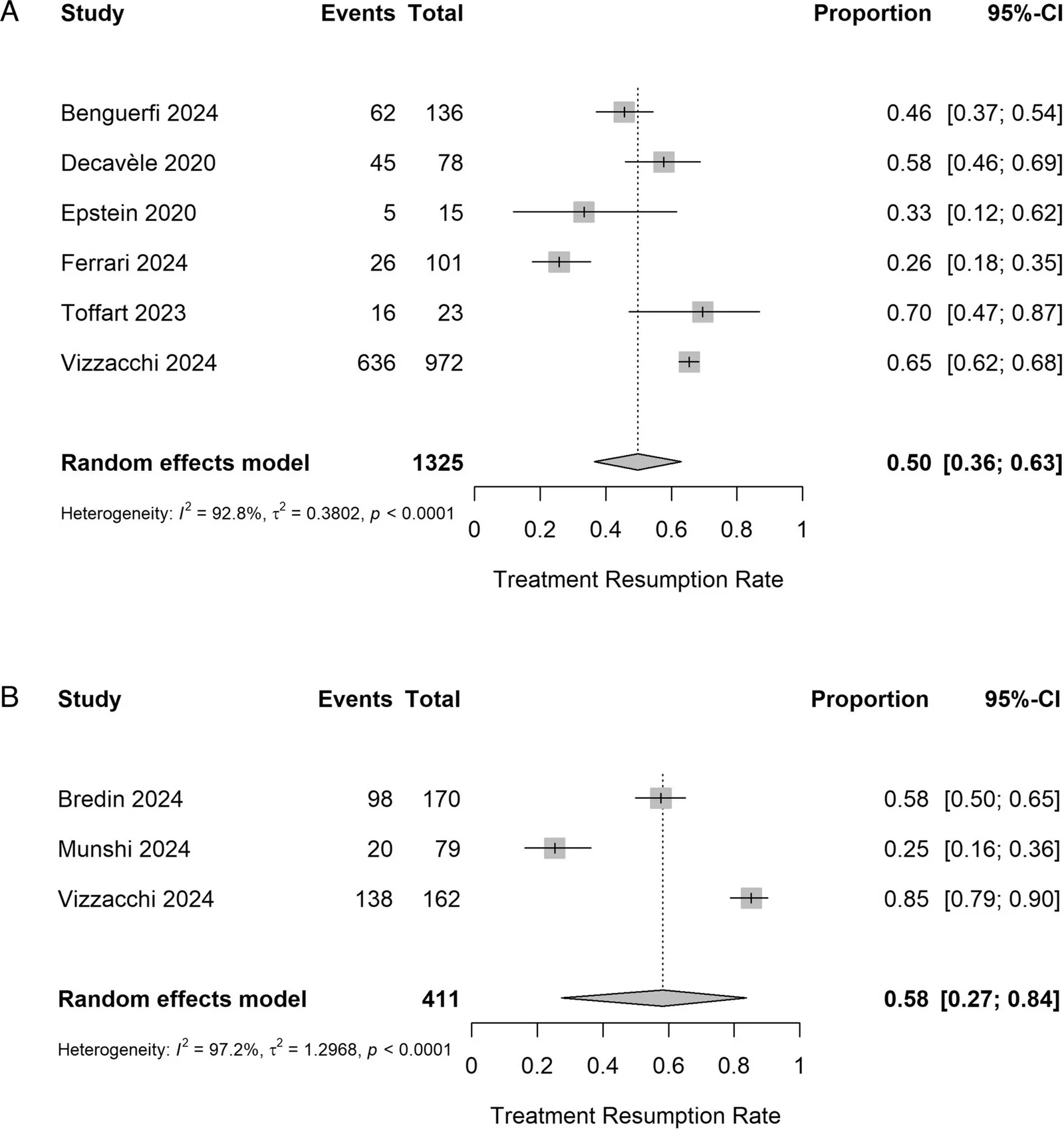
Random-effects forest plot of resumption of the original anticancer treatment after ICU admission in patients with solid (**A**) and hematological malignancies (**B**)

We assessed the separate data from patients with solid and hematological malignancies from one study [10]. The pooled treatment resumption rate from 2772 patients with solid malignancies was 60% (95% CI, 49–71%) within 6 months after discharge (Fig. 3A). Among 423 patients with hematological malignancies, the pooled resumption of treatment was 77% (95% CI, 56–89%) (Fig. 3B). The resumption of the original treatment was 50% (95% CI, 36–63%) for patients with solid malignancies (*N* = 1325) (Fig. 4A) and 50% (95% CI, 27–84%) for patients with hematological malignancies (*N* = 411) (Fig. 4B). All analyses had substantial heterogeneity (Fig. 3).

### Risk Factors

Six studies assessed risk factors associated with resuming anticancer treatment. Performance status (at admission [29, 31] or worse at discharge compared with admission [10, 11]) was associated with lower odds of resuming treatment in three studies. Two studies [12, 29] reported age as a variable associated with discontinuation of anticancer treatment. Other risk factors for discontinuation or modification of treatment included: delirium [10], hyperbilirubinemia [12], more than one organ failure [25], therapeutic limitations [12, 25], cancer progression at ICU admission [21, 31], corticosteroid use at ICU admission [21], and gastrointestinal tract or lung site among solid tumors [31].

## Discussion

In this systematic review and meta-analysis, we identified 20 studies (3531 patients) that evaluated the resumption of anticancer treatment after unplanned ICU admission. The pooled rate of resuming any anticancer treatment was 61%, while the rate of returning to the original planned therapy was 46%. Interestingly, both rates appeared slightly higher in patients with hematological malignancies compared to those with solid tumors. Our findings were limited by heterogeneity across cancer types and study designs.

Patients with cancer represent a substantial and growing proportion of the ICU population [6]. Most previous studies have focused primarily on organ support and short-term outcomes [7, 33]. Nevertheless, the question of whether these patients can subsequently receive anticancer treatment has received far less attention. Beyond survival, understanding whether patients with cancer return to treatment remains a clinically meaningful question for both patients and oncologists [34], as the continuation of anticancer treatment is a primary determinant of long-term survival [10–12, 29]. The observed variability in resumption rates likely reflects both clinical and methodological heterogeneity. Nearly all included studies were single-center, and treatment continuation decisions are inherently influenced by local practice patterns. Furthermore, the included patients varied widely in tumor type and site, disease stage, and ICU admission thresholds. Patient preferences and factors that affected whether patients returned to treatment were also not reported in many studies. Several studies reported the number of patients who received any systemic anticancer treatment after discharge, but did not report whether those who did not receive treatment had previously planned treatments. These limitations contribute to the observed statistical heterogeneity.

The finding that patients with hematological malignancies had higher rates of treatment resumption deserves consideration. Potential explanations include admission due to critical illness secondary to the hematological malignancies, which may respond to urgent treatments [35], and different post-ICU treatment decisions by hematologic and solid tumor oncology specialists [36]. However, given the substantial heterogeneity among the included studies, the differences in treatment resumption rates between patients with solid and those with hematological cancer require confirmation in larger prospective cohorts.

This review has several strengths. We conducted a comprehensive search across multiple databases without language restrictions and distinguished between resuming any anticancer therapy and continuing the originally planned regimen, which are two clinically distinct outcomes with different prognostic implications. However, our review also has important limitations. The small number of eligible studies and the predominance of single-center, retrospective designs, as well as variable thresholds for ICU admission, lack of information surrounding specific reasons for treatment modifications (such as functional status, patient preference, disease progression), heterogeneity with respect to cancer subtype and treatments administered, precluded more detailed subgroup analyses, and limited our ability to explore sources of heterogeneity in depth. The lack of a uniform definition of treatment resumption across studies introduces conceptual heterogeneity that is difficult to fully account for in a meta-analysis. Additionally, the studies did not compare patients admitted to the ICU with patients admitted to the hospital who did not require ICU admission. Thus, we can’t infer whether treatment resumption rates were a consequence of ICU admission or of hospitalization more broadly. However, at least one previous study suggested that cancer progression was higher among patients who experienced an ICU admission during their hospitalization than among those who did not [37]. A lower treatment resumption rate may be one of the factors related to this finding. Our findings carry important implications for both intensivists and oncologists, who usually have different views on critically ill patients with cancer[38]. For intensivists, the data suggest that ICU admission does not preclude subsequent anticancer therapy in a substantial proportion of survivors, supporting the potential value of critical care in patients with curable or treatment-responsive malignancies. For oncologists, awareness that more than half of patients may not resume their originally planned treatment highlights the need for early post-ICU reassessment of disease status, functional capacity, and patient preferences. Future research should prioritize prospective, multicenter studies with standardized definitions of treatment resumption, predefined assessment time points, and integration of functional and patient-reported outcomes.

In conclusion, anticancer treatment is resumed in most patients who survive an ICU admission, but continuation of the original treatment plan occurs in fewer than half. Thus, ICU admission may represent a turning point in the oncologic trajectory and should prompt careful reassessment after discharge.

## Supplementary Information

Below is the link to the electronic supplementary material.

ESM 1 (DOCX 2.82 MB)

## Data Availability

Data and material are available upon request.

## Declarations

### Declaration of AI-assisted technologies in the writing process

During the preparation of this work, the authors used Grammarly (Grammarly, San Francisco, USA) to revise the English language. After using this tool, the authors reviewed and edited the content as needed and take full responsibility for the manuscript.
