# Supplementary material for: Impact of intensive care unit admission on the resumption of treatment in patients with cancer: a systematic review and meta-analysis
Source: Support Care Cancer. 2026 Aug 31;34(9):915. doi: 10.1007/s00520-026-11135-6 (PMC13529920; doi:10.1007/s00520-026-11135-6)
Supplement: Supplementary file 1 [file 520_2026_11135_MOESM1_ESM.docx]

**Impact of Intensive Care Unit Admission on the Resumption of Treatment in Patients with Cancer: A Systematic Review and Meta-Analysis**

Raisa do Val Roso

Renato Daltro de Oliveira

Laveena Munshi

Antonio Paulo Nassar Junior

**Supplementary material**

Table S1. Search strategies and results by database (conducted on February 24, 2025)

| **Database** | **Search Strategy** | **Results** |
| --- | --- | --- |
| **PubMed** | ("malignancy"[Title/Abstract] OR "malignancies"[Title/Abstract] OR "cancer"[Title/Abstract]) AND ("Intensive Care"[All Fields] OR "ICU"[All Fields] OR "critically ill"[All Fields] OR "critical illness"[All Fields]) AND ("anticancer treatment"[All Fields] OR "anticancer therapy"[All Fields] OR "cancer treatment"[All Fields] OR "cancer therapy"[All Fields] OR "long-term outcomes"[All Fields]) | 875 |
| **Embase** | ('cancer':ti,ab,kw OR 'malignancy':ti,ab,kw OR 'malignancies':ti,ab,kw) AND ('intensive care':ti,ab,kw OR 'icu':ti,ab,kw OR 'critically ill':ti,ab,kw OR 'critical illness':ti,ab,kw) AND ('cancer treatment':ti,ab,kw OR 'anticancer treatment':ti,ab,kw OR 'cancer therapy':ti,ab,kw OR 'anticancer therapy':ti,ab,kw OR 'long-term outcomes':ti,ab,kw) | 1065 |
| **Scopus** | (TITLE-ABS-KEY('cancer' OR 'malignancy' OR 'malignancies') AND TITLE-ABS-KEY('intensive care' OR 'icu' OR 'critically ill' OR 'critical illness') AND TITLE-ABS-KEY('cancer treatment' OR 'anticancer treatment' OR 'cancer therapy' OR 'anticancer therapy' OR 'long-term outcomes')) | 287 |
| **Web of Science** | ((TI=(("cancer" OR "malignancy" OR "malignancies") AND ("ICU" OR "intensive care" OR "critically ill" OR "critical illness")) OR AB=(("cancer" OR "malignancy" OR "malignancies") AND ("ICU" OR "intensive care" OR "critically ill" OR "critical illness"))) AND AB=(("cancer treatment") OR ("anticancer treatment") OR ("cancer therapy") OR ("anticancer therapy") OR ("long-term outcomes"))) | 370 |
| **Total** |  | **2597** |

Table S2. Risk of Bias Summary (Joanna Briggs Institute Critical Appraisal for Studies Reporting Prevalence Data)

|  | 1 | 2 | 3 | 4 | 5 | 6 | 7 | 8 | 9 | Overall Appraisal |
| --- | --- | --- | --- | --- | --- | --- | --- | --- | --- | --- |
| Roques, 2009 | No | Yes | No | Yes | Yes | Yes | Yes | Yes | Yes | 6 |
| Andréjak, 2011 | No | Yes | No | Yes | Yes | Yes | Yes | Yes | Yes | 7 |
| Soares, 2014 | Yes | Yes | Yes | Yes | Yes | No | Yes | Yes | No | 7 |
| Lee, 2015 | No | Yes | Yes | Yes | Yes | Yes | Yes | Yes | Yes | 8 |
| Wohlfarth, 2015 | No | Yes | No | Yes | Yes | Yes | Yes | Yes | No | 6 |
| Auclin, 2017 | No | Yes | Yes | Yes | Yes | Yes | Yes | Yes | Yes | 8 |
| Decavèle,2020 | No | Yes | No | Yes | Yes | Yes | Yes | Yes | Yes | 7 |
| Epstein, 2020 | No | Yes | No | Yes | Yes | Yes | Yes | Yes | Yes | 6 |
| Gheerbrant, 2021 | No | Yes | Yes | Yes | Yes | Yes | Yes | Yes | Yes | 8 |
| Vigneron, 2021 | No | Yes | Yes | Yes | Yes | Yes | Yes | Yes | No | 7 |
| Hautecloque-Rayz, 2022 | No | Yes | Yes | Yes | Yes | No | Yes | Yes | Yes | 7 |
| Toffart, 2023 | No | No | No | Yes | Yes | Yes | Yes | Yes | Yes | 6 |
| Vigneron, 2023 | No | Yes | Yes | No | Yes | Yes | Yes | Yes | No | 6 |
| Benguerfi, 2024 | No | Yes | Yes | Yes | Yes | Yes | Yes | Yes | Yes | 8 |
| de Herreros, 2024 | No | Yes | No | Yes | Yes | Yes | Yes | Yes | No | 6 |
| Ferrari, 2024 | No | Yes | No | Yes | Yes | Yes | Yes | Yes | No | 6 |
| Lee, 2024 | No | Yes | Yes | Yes | Yes | Yes | Yes | Yes | Yes | 8 |
| Vizzacchi, 2024 | No | Yes | Yes | Yes | Yes | Yes | Yes | Yes | Yes | 8 |
| Bredin, 2024 | No | Yes | Yes | Yes | Yes | Yes | Yes | Yes | Yes | 8 |
| Munshi, 2024 | Yes | Yes | Yes | Yes | Yes | Yes | Yes | Yes | Yes | 9 |

Domains:

1. Was the sample frame appropriate to address the target population?
2. Were study participants sampled in an appropriate way?
3. Was the sample size adequate?
4. Were the study subjects and the setting described in detail?
5. Was the data analysis conducted with sufficient coverage of the identified sample?
6. Were valid methods used for the identification of the condition?
7. Was the condition measured in a standard, reliable way for all participants?
8. Was there appropriate statistical analysis?
9. Was the response rate adequate, and if not, was the low response rate managed appropriately?

Table S3. Description of the definitions and findings of resumption of anticancer therapy among included studies

| Study | Year | Methods: Definitions of the anticancer resumption | Results: Description of the anticancer treatment resumption |
| --- | --- | --- | --- |
| Roques | 2009 | “The administration of specific anticancer treatments after discharge, i.e., chemotherapy, radiation therapy and surgery, as well as the vital status at 6 months were recorded.” | “Thirty patients (68%) of the 44 ICU survivors for whom clinical information was available were able to receive specific anticancer treatment after hospital discharge.” |
| Andréjak | 2011 | “We recorded the following data on management in the MICU: (…) the anti-cancer treatments.” | “Four patients underwent lung cancer treatment after MICU discharge.” |
| Soares | 2014 | “All patients were followed up  until hospital discharge. In addition, hospital survivors were followed up  until 6 months after ICU admission and information was assessed during  clinical consultations or phone calls.” | “Anticancer treatments were recommended to 108 (39%) hospital survivors and administered to 102; anticancer treatment was not recommended to 121 (44%) patients and information on this item was not available for 46 (17%) patients.” |
| Lee | 2015 | “We also evaluated whether additional treatments such as chemotherapy or radiotherapy and its compliance with pre-  planned treatment for each cancer could be delivered after survival discharge from the ICU.” | “Additional treatment for cancer could be delivered in 161 of 356 patients (45.2%) discharged from the ICU.” |
| Wohlfarth | 2015 | “we recorded start dates and types of immuno/chemotherapies, hematologic outcomes, any unscheduled ICU admission, date of death (if applicable), and last visit.” | “All but three ICU survivors with newly diagnosed DLBCL (75%) received full-dose immuno-/chemotherapy  after ICU discharge.”^1^ |
| Auclin | 2017 | “anticancer treatment resumption was also assessed” | “Out of the 146 patients with advanced disease who theoretically had an indication for additional cancer therapy, 77 (52.7%) received treatment, 54 (37.0%) did not receive treatment, and 15 patients (10.3%) were lost to follow-up.” |
| Decavèle | 2020 | “Anticancer therapy after ICU discharge was classified as follows: (1) continued, when the anticancer therapy planned and initiated before ICU admission was continued unchanged after ICU discharge, (2) modified, when the anticancer therapy planned  and initiated before ICU admission was changed or stopped after ICU discharge, and (3) initiated, when, for patients with newly diagnosed cancer, anticancer therapy was initiated during or after the ICU stay. Anticancer therapy only comprised chemotherapy and radiation therapy.” | “Among the 78 ICU survivors, anticancer therapy was continued in 32 (41%) patients, modified in 33 (42%) patients, and initiated in 13 (17%) patients.” |
| Epstein | 2020 | “We performed an analysis with the purpose of identifying the extent of clinical benefit derived from posthospital discharge chemotherapy in this specific subgroup of patients.”  “We also analyzed receipt and benefit from nonchemotherapy  cancer treatments such as liver-directed therapies.” | “Of the 15 patients surviving to hospital discharge, 6 (21%)  received further chemotherapy after discharge, but none showed  evidence of clinical benefit.” |
| Gheerbrant | 2021 | “Data regarding ECOG-PS and new anticancer treatments administered were collected at 3- and 6-months following ICU discharge.” | “At 3 months post-ICU discharge, 74 (55%) patients received anticancer treatments.” |
| Vigneron | 2021 | Detailed information provided by the authors | Among the 157 patients discharged alive from **hospitalization** (not from ICU):  - 33 patients did not continue their treatment despite a valid indication for it, due to a deteriorated general condition  - 58 patients did continue it, but I have no information regarding any possible modification of the therapeutic regimen  - 24 patients did not continue it since there was no indication to pursue it before their admission to ICU  - For 42 patients, we have no information about treatment resumption. |
| Hautecloque-Rayz | 2022 | “Patients who did not receive systemic treatment were censored at the date of the last follow-up.” | “Out of 108 patients discharged from the ICU, data about (re)starting of systemic treatment were available  for 96 patients (missing data for 12 cases). Among these 96 patients, 59 have received systemic treatment after  ICU discharge (61%).” |
| Toffart | 2023 | “The continuation or discontinuation of anticancer treatment at 3 months was also determined.” | “At 3 months, for 16/23 (70%) ICU patients, cancer care  was not modified compared to baseline.” |
| Vigneron | 2023 | “The following characteristics of breast cancer were collected: (…) treatment within three months prior to ICU admission (surgery, radiotherapy, chemotherapy, endocrine therapy, targeted therapy including trastuzumab, pertuzumab, bevacizumab, lapatinib, erlotinib, sacituzumab-govitecan, atezolizumab and palbociclib)” | “The features of anti-tumoral treatment after hospital discharge could be obtained for 84 of 96 ICU survivors previously treated with antitumoral treatment or newly diagnosed. Antitumoral treatment was continued for 65 of them (77.4%)”. |
| Benguerfi | 2024 | “After the ICU stay, we collected the following  data: (…), cancer treatment administration (any systemic or local cancer treatment introduced after the ICU stay) and its type (chemotherapy, surgery, radiotherapy, immunotherapy, targeted therapy and/or hormotherapy), treatment adjustment (protocol chosen due  to expected lower toxicity, dose reduction, early discontinuation of the treatment).” | “Among the 136 patients who survived the ICU stay, 81 (59.6%) received cancer treatment after ICU discharge.  The main treatments were chemotherapy (n=32 [39.5%]), surgery (n=11 [13.6%]), radiotherapy (n=9 [11.1%]), hormonotherapy (n=7 [8.6%]) or immune checkpoint inhibitors (n=5 [6.2%]). A treatment adjustment was made for 19 (30.2%) patients.” |
| de Herreros | 2024 | “We assessed data regarding the patients’ conditions at discharge and clinical outcomes, such as modifications to or discontinuations of cancer treatment.” | “Oncological treatment was definitively discontinued in 40% of the patients, whereas dose reduction or delay in treatment was necessary in 56.7% of patients. In 18.6% of the cases, treatment was changed due to cancer progression or clinical deterioration.” |
| Lee | 2024 | “Among ICU survivors, whether  chemotherapy was restarted after ICU discharge and causes of in-hospital death without ICU re-admission were collected.” | “Among patients (…) who survived in the ICU (n = 568), 246 (45.9%) patients received chemotherapy after ICU discharge.” |
| Vizzacchi^2^ | 2024 | “We defined change of treatment if any of the previous planned cancer treatment before ICU admission was discontinued.” | “The change of cancer treatment occurred in 360 patients (31.7%).” |
| Bredin | 2024 | “We recorded the characteristics of cancer treatment after the ICU stay (drug regimens and doses) and reasons for eventual changes in the intended therapeutic regimen.” | “Following ICU discharge, 72 patients (42.3%) required some modifications of hematologic treatment regimens,  including full discontinuation in 39 patients (22.9%, including 30 patients with AML and 9 patients with B-cell NHL), drug changes in 15 patients (8.8%), delayed administration in 11 patients (6.4%), and dose reduction in 7 patients (4.1%).” |
| Munshi | 2024 | “Among patients who had not completed the full course of  their cancer treatment at the time of ICU admission, we  evaluated “return to treatment” defined as receipt of any  cancer treatment within 6 months. We further evaluated whether the post-ICU treatment was modified from the original planned treatment (e.g., dose reduction, medication modification).” | “Across those alive at 6 months, 74% (n=79/107) reported their treatment status (after removing unknown  or patients who did not have any pending treatment). Of these, 25% (20/79) continued with planned treatment, 23% (18/79) had a modification to their treatment plan (dose reduced, modified, palliative intent), and 39% (31/79) were no longer candidates for treatment.” |
| Ferrari | 2025 | “Data for all surviving patients were time-censored at the 12th-  month or at the 60th month based on each institution’s  electronic records. Subsequent lines of treatment referred  to either chemotherapy, targeted cancer therapies and/or  immunotherapy.” | “Antineoplastic treatments resumed in 40 (30%) patients but treatment was intensified in only 14 cases.” |

^1^ There were 12 patients with newly diagnosed diffuse large B-cell lymphoma among ICU survivors in the study by Wohlfarth et al.

^2^ We asked for additional data on the complete suspension of anticancer treatment in the study of Vizzacchi et al.

Figure S1. Funnel plot of the included studies
